# The effects of omega-3 polyunsaturated fatty acids on muscle and whole-body protein synthesis: a systematic review and meta-analysis

**DOI:** 10.1093/nutrit/nuae055

**Published:** 2024-05-23

**Authors:** Atiporn Therdyothin, Konstantinos Prokopidis, Francesco Galli, Oliver C Witard, Masoud Isanejad

**Affiliations:** Department of Musculoskeletal and Ageing Science, University of Liverpool, Liverpool, L7 8TX, United Kingdom; Department of Orthopedics, Police General Hospital, Bangkok, Pathum Wan, Bangkok, 10330, Thailand; Department of Musculoskeletal and Ageing Science, University of Liverpool, Liverpool, L7 8TX, United Kingdom; Department of Pharmaceutical Sciences, Università degli Studi di Perugia, Perugia, Piazza dell'Università, 1, Perugia PG, 06123, Italy; Centre of Human & Applied Physiological Research, King’s College London, London, SE1 1UL, United Kingdom; Department of Musculoskeletal and Ageing Science, University of Liverpool, Liverpool, L7 8TX, United Kingdom

**Keywords:** omega-3, fish oil, sarcopenia, muscle protein synthesis, fractional synthetic rate

## Abstract

**Context:**

Sarcopenia describes the age-related decline in skeletal muscle mass and strength that is driven, at least in part, by an imbalance between rates of muscle protein synthesis (MPS) and muscle protein breakdown. An expanding body of literature has examined the effect of omega-3 polyunsaturated fatty acid (n-3 PUFA) ingestion on MPS rates in older adults, with mixed findings.

**Objective:**

The aim of this systematic review and meta-analysis was to investigate the effectiveness of n-3 PUFA ingestion in stimulating rates of MPS and whole-body protein synthesis in healthy adults and clinical populations.

**Data Sources:**

Searches were conducted of the PubMed, Web of Science, Cochrane Library, and Scopus databases from inception until December 2022 for articles on randomized controlled trials comparing the effect of n-3 PUFA ingestion vs a control or placebo on rates of MPS and whole-body protein synthesis. The search yielded 302 studies, of which 8 were eligible for inclusion.

**Data Extraction:**

The random effects inverse-variance model was used and standardized mean differences (SMDs) with 95%CIs were calculated to assess the pooled effect. Risk of bias was assessed by the Cochrane Risk‐of‐Bias 2 tool.

**Data Analysis:**

The main analysis indicated no effect of n-3 PUFA supplementation on MPS rates (k = 6; SMD: 0.03; 95%CI, −0.35 to 0.40; I^2^ = 30%; P = .89). Subgroup analysis based on age, n-3 PUFA dose, duration of supplementation, and method used to measure fractional synthetic rate also revealed no effect of n-3 PUFA ingestion on MPS. In contrast, the main analysis demonstrated an effect of n-3 PUFA ingestion on increasing whole-body protein synthesis rates (k = 3; SMD: 0.51; 95%CI, 0.12–0.90; I^2^ = 0%; P = .01).

**Conclusions:**

n-3 PUFA ingestion augments the stimulation of whole-body protein synthesis rates in healthy adults and clinical populations.

**Systematic Review Registration:**

PROSPERO registration no. 42022366986.

## INTRODUCTION

Sarcopenia describes the age-associated decline in skeletal muscle strength, quality, and mass,^1^ and is estimated to impact more than 50 million people worldwide.^2^ This clinical condition affects musculoskeletal function, thus increasing the likelihood of falls and fractures with advancing age.^3^^,^^4^ Sarcopenia also leads to a decline in overall physical health and increased risk of cardiovascular disease,^5^ dependence on others,^6^ and, eventually, death.^7^

At the metabolic level, skeletal muscle proteins are in a constant and dynamic state of turnover, oscillating between fasted losses in muscle protein and fed-state gains in muscle protein. In the fasted state, muscle protein breakdown (MPB) exceeds muscle protein synthesis (MPS), leading to a net loss of muscle protein. Conversely, a net gain in muscle protein ensues in the fed state when rates of MPS exceed MPB.^8^ The measurement of in vivo MPB is technically challenging; MPS is commonly measured in metabolic studies to represent the acute muscle anabolic response to nutritional stimuli and is expressed as fractional synthetic rate (FSR).^9^

Various nutritional strategies have been investigated in terms of efficacy to stimulate MPS and promote muscle mass,^10–12^ including omega-3 polyunsaturated fatty acid (n-3 PUFA) supplementation.^13^ However, clinical studies regarding the efficacy of n-3 PUFA supplementation to increase MPS have yielded conflicting results, which primarily have been attributed to the heterogeneity in study population, design, and outcome measurements. Early studies by Smith et al^13^^,^^14^ demonstrated that n-3 PUFA supplementation increased the MPS response to anabolic stimuli in healthy young individuals and older adults. Other studies have demonstrated an effect of n-3 PUFA supplementation in stimulating MPS and attenuating muscle atrophy during a simulated catabolic situation, such as leg immobilization.^15^ Conversely, some studies have shown no statistical effect of n-3 PUFA ingestion on MPS rates in young healthy men under resting and postexercise conditions.^16^ Therefore, the purpose of this systematic review and meta-analysis of clinical trials is to examine the impact of n-3 PUFA supplementation on MPS. We hypothesized that the preponderance of studies would demonstrate a positive effect of n-3 PUFA supplementation in increasing protein synthesis rates at the whole-body and muscle levels in healthy adults and clinical populations.

## METHODS

This systematic review and meta‐analysis were conducted according to the Preferred Reporting Items for Systematic Reviews and Meta‐Analyses (PRISMA) guidelines.^17^ The protocol was registered in the International Prospective Register of Systematic Reviews (PROSPERO; identifier 42022366986).

### Search strategy

The PubMed, Web of Science, Cochrane Library, and Scopus databases were searched by 2 independent reviewers (K.P. and A.T.) for relevant studies from inception until December 2022. The search strategy used in each database is described in Table S1. No restrictions in terms of geographic region or language were applied. A manual search of references and published reviews was also conducted. Two reviewers independently assessed the titles and abstracts against the inclusion and exclusion criteria. Full-text articles of potentially eligible studies were retrieved and screened independently. Any disagreements in the literature search process were resolved by a third investigator (M.I.).

Studies were included on the basis of the following criteria: (1) randomized and nonrandomized controlled trials (RCTs); (2) included healthy individuals or individuals with comorbidities irrespective of age; (3) intervention included n-3 PUFA supplementation; and (4) n-3 PUFA supplementation was administered either as a combined intervention with other nutrition supplementation or physical exercise when an appropriate control group was included. Published articles were excluded if they (1) were in vitro studies; (2) involved enteral nutrition; (3) did not contain primary data (ie, reviews, letters, or commentaries); and (4) were not published as a full text. The PICOS framework for inclusion of studies is depicted in Table S2.

### Data extraction and risk of bias

Two authors (K.P. and A.T.) independently extracted all data sets that included the name of the first author, date of publication, country of origin, number of participants, description of the intervention involving n-3 PUFA supplementation, description of the control or comparator, the assessment method(s) of MPS, body composition, and n-3 PUFA intake. Discrepancies among obtained data sets were resolved by discussion with another team member (M.I.). The quality of each study was independently evaluated using the Cochrane Risk‐of‐Bias 2 (RoB2) tool by 2 reviewers (K.P. and A.T.) based on a randomization process, deviations from intended interventions, missing outcome data, outcome measurement, and selective reporting of outcomes.^18^^,^^19^ Studies were then categorized as having low, moderate, or high risk of bias.

### Statistical analysis

Quantitative data were analyzed as continuous variables. Using Review Manager (RevMan 5.4.1; Cochrane.org) software, mean differences were calculated from the change in outcomes after receiving supplementation or placebo. Standardized mean differences (SMDs) were calculated when it was not possible to convert units for measurements of the same outcome. Graphical values were extracted using DigitizIt 2.5 software (Bormisoft, Braunschweig, Germany) when it was not possible to obtain numeric data. Given the anticipated heterogenous nature of the data set, random effects model and inverse‐variance methods were applied in the analysis of pooled results. Missing SDs for measured outcomes were estimated from CIs, SEs, *t* and *P* statistical values, or by imputing SDs from similar studies.

Statistical heterogeneity of study outcomes was assessed using the overlap of their 95%CI and expressed as measurements of Cochrane’s *Q* (χ^2^ test). The *I*^2^ statistic was used to quantify the heterogeneity among studies, classifying pooled data as exhibiting moderate heterogeneity when *I*^2^ ranged from 50% to 74% and high heterogeneity when *I*^2^ < 75%.^20^ Sensitivity analyses were performed to evaluate the robustness of the pooled results by disregarding the effect of additional interventions (eg, other nutritional interventions, physical exercise interventions) on outcome measurements, excluding studies with a high risk of bias, and studies that did not assess background dietary n-3 PUFA intake. Subgroup analyses were performed on the bases of age, health status, dose, and treatment duration. Publication bias was not assessed using a funnel plot, because of the limited number of studies included in the meta-analysis (*k* < 10).

## RESULTS

**Figure 1.**
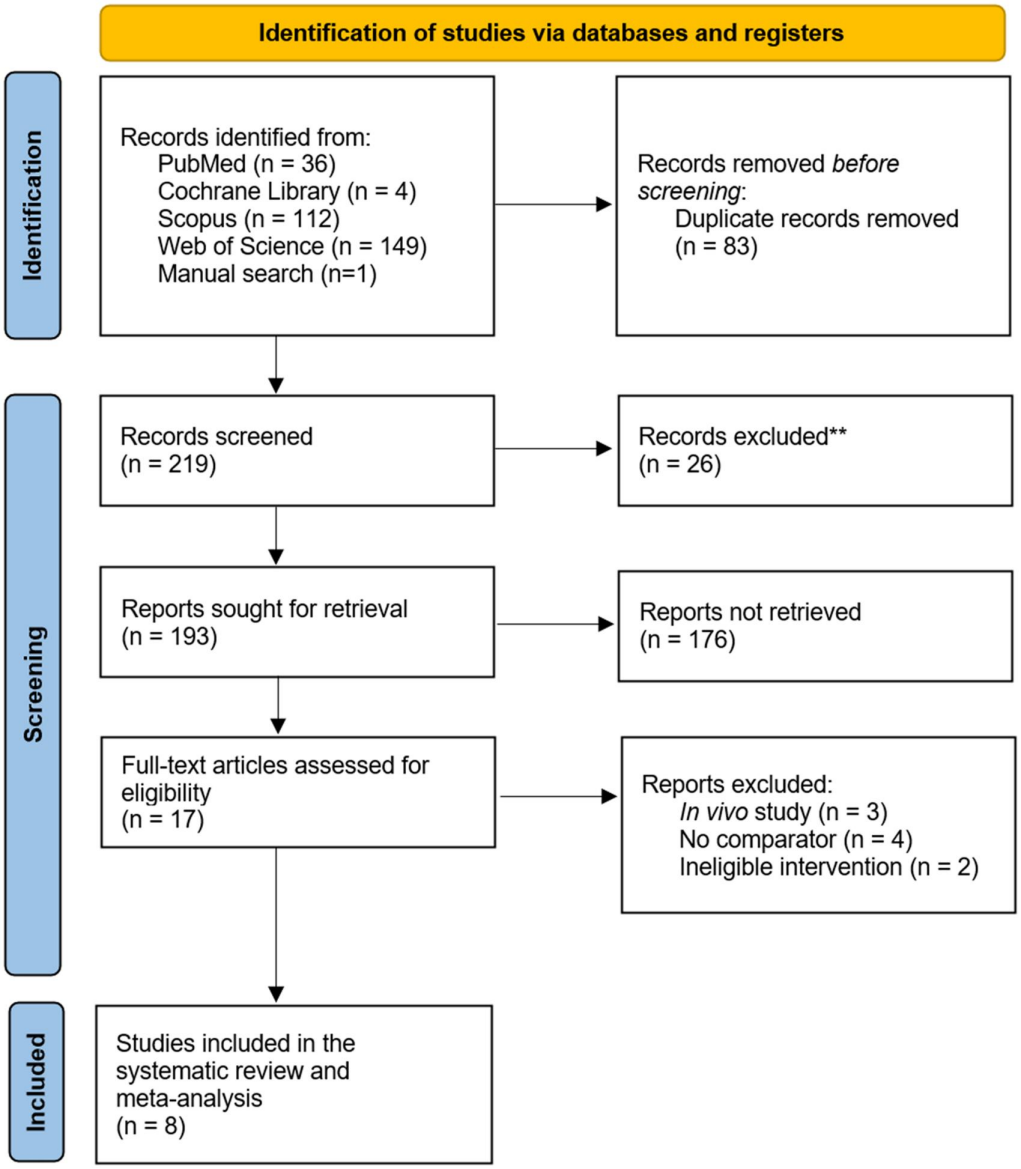
Preferred Reporting Items for Systematic Reviews and Meta‐Analyses (PRISMA) flow diagram.

The literature search identified 302 publications. After 82 duplicates and 26 irrelevant documents were removed, 193 articles were retrieved. After screening of titles and abstracts, 176 articles were deemed irrelevant, and full-text articles reporting on a total of 17 studies were screened. Of these publications, 3 were in vivo studies,^21–23^ 4 had no control or baseline measurement of MPS,^16^^,^^24^^,^^25^ and 2 did not exclusively contain n-3 PUFA in their PUFA supplement formula.^26^^,^^27^ In total, 8 studies that investigated the effect of n-3 PUFA ingestion on MPS were included in the systematic review and meta-analysis (Figure  1).^13^^,^^15^^,^^28–33^

Characteristics, study population, and primary outcome measurements are summarized in Table 1. Of the included studies, 4 were conducted in the United States,^13^^,^^28^^,^^31^^,^^33^ 2 in the United Kingdom,^30^^,^^32^ 1 in Canada,^15^ and 1 in Ireland.^31^ The preponderance of studies recruited healthy participants, with 5 studies conducted with medically stable older adults aged 50 years or older,^13^^,^^28^^,^^30–32^ and 1 study with young healthy women.^15^ Only 2 studies were conducted with individuals with chronic diseases, such as chronic obstructive pulmonary disease (COPD),^29^ and patients with end-stage renal disease who were undergoing maintenance hemodialysis.^33^ Five studies consisted of a cohort of both men and women,^28^^,^^29^^,^^31–33^ and 2 trials were conducted only with women.^15^^,^^30^

**Table 1.** Study and Participant Characteristics of the Included Studies in the Meta-Analysis

Study, year	Country	Study design	Health status	Total	n-3		Comparator		Treatment dose (g/d)	Treatment duration	Additional intervention	Reported outcomes	MPS measurement (duration)
				No. (M/F)	No. (M/F)	Age (SD), y	No. (M/F)	**Age** **(SD)**					
Kunz, 2022^28^	USA	Double-blind RCT	Independently living older adults	63 (29/34)	30 (16/14)	71.4 (4.5)	33 (13/20)	71.5 (4.8)	3.9 g (2.7 g EPA, 1.2 g DHA)	6 mo	–	MPS (mixed FSR, whole-body protein synthesis, protein balance), muscle strength (knee extension 1-RM), muscle quality (1-RM/lean leg mass), muscle mass (lean leg mass), performance (peak oxygen consumption)	l-phenylalanine (4 h)
Murphy, 2021^31^	Ireland	Double-blind RCT	Urban, community-dwelling older adults	76 (35/41)	38 (17/21)	73 (6)	38 (18/20)	70 (5)	4.8 g (1.56 g EPA, 2.28 g DHA)	24 wk	21.2 g protein (6.2 g leucine/d)	MPS (myofibrillar), muscle strength (handgrip, knee flexion torque, knee extension torque), muscle mass (appendicular lean mass), performance (SPPB, gait speed, 5 times sit to stand, timed up-and-go, single leg standing balance)	Deuterium-labelled alanine (72 h)
Brook, 2021^30^	UK	Within-subject, double-blind, placebo-controlled trial	Healthy, recreationally active older women	16 (0/16)	8 (0/8)	64.4 (0.8)	8 (0/8)	66.5 (1.4)	3.680 g (1.860 g EPA, 1.540 g DHA)	6 wk	Unilateral resistance exercise training 3 times/wk both groups	MPS (myofibrillar FSR, ASR), muscle strength (knee extension 1-RM, MVC), lean body mass, muscle mass (thigh fat-free mass), muscle CSA	Deuterium-labelled alanine (2 wk)
Engelen, 2022^29^	USA	Double-blind RCT	Patients with COPD grade II-IV	32 (18/14)	10 (5/5) (low dose) 12 (6/6) (high dose)	70 (7.85) (low dose) 67.58 (7.48) (high dose)	10 (7/3)	62.10 (11.09)	2 g (1.2 g EPA + 0.8 g DHA) + 3 g olive oil (low dose) 3.5 g (2.1 g EPA + 1.4 g DHA) (high dose)	4 wk	0.06 g/kg fat-free mass high-quality hydrolyzed casein protein both groups in fed state	MPS (whole body protein synthesis, net protein synthesis), MPB (net protein breakdown), muscle strength (handgrip), lean body mass, muscle mass (lean mass extremities), performance (physical activity, maximal inspiratory pressure, quality of life score)	l-phenylalanine (4-h fasting state, 40 min fed)
McGlory, 2019^15^	Canada	Double-blind RCT, repeated measures study	Healthy, recreationally active young women	20 (0/20)	11 (0/11)	22 (3)	9 (0/9)	22 (3)	2.97 g EPA, 2.03 g DHA	8 wk	Leg immobilization for 2 wk (weeks 4-6) then return to normal activity (weeks 6-8)	MPS (myofibrillar FSR), muscle strength (knee extension isometric torque), muscle mass (lean leg mass), muscle volume (quadriceps), muscle CSA (quadriceps)	Deuterium-labelled alanine (3 d baseline, 14 d for immobilization and recovery)
Da Boit, 2017^32^	UK	Double-blind RCT	Medically stable older adults	50 (27/23)	27 (14/13)	70.1 (3.6)	23 (13/10)	73 (5.0)	3 g (2.1 g EPA, 0.6 g DHA)	18 wk	Resistance exercise twice a week (leg press, leg extension, leg curl, calf press) both groups	MPS (myofibrillar FSR), muscle strength (knee extension torque), muscle CSA (quadriceps), muscle quality (knee-extensor isometric strength per unit CSA), performance (4-min walk time, chair-rise time)	Deuterium-labelled alanine (96 h)
Deger, 2016^33^	USA	Double-blind RCT	Patients receiving maintenance hemodialysis who have chronic inflammation	20 (17/3)	11 (9/2)	53 (9)	9 (8/1)	53(13)	2.9 g (2:1 ratio of EPA to DHA)	12 wk	–	MPS (whole-body protein synthesis, forearm protein synthesis, net protein balance), MPB (whole body protein breakdown, forearm protein breakdown)	l-phenylalanine (3 h)
Smith, 2011^13^	USA	Single-blinded RCT	Healthy older adults	15 (10/5)	8 (5/3)	71 (1)	7 (5/2)	71 (1)	4 g (1.86 g EPA, 1.50 g DHA)	8 wk	Hyperaminoacidemic-hyperinsulinemic clamp for 3 h in both groups	MPS (mixed FSR)	l-phenylalanine (3-h basal FSR, 2.5-h hyperaminoacidemic-hyperinsulinemic state)

*Abbreviations*: ASR, absolute synthetic rate; COPD, chronic obstructive pulmonary disease; CSA, cross-sectional area; DHA, docosahexaenoic acid; EPA, eicosapentanoic acid; FSR, fractional synthetic rate; MPB, muscle protein breakdown; MPS, muscle protein synthesis; MVC, maximal voluntary contraction; RCT, randomized controlled trial; SPPB, short physical performance battery; UK, United Kingdom; USA, United States of America; 1-RM, 1-repetition maximum.

All included studies were RCTs. Seven studies were double-blinded RCTs,^15^^,^^28–33^ and 1 study was single-blinded.^13^ The dose of n-3 PUFA supplementation was ≤3 g/d in 3 studies,^15^^,^^32^^,^^33^ >3 g/d in 4 studies.^13^^,^^28^^,^^30^^,^^31^ Another study compared both a lower and higher dose of n-3 supplementation to placebo.^29^ In 1 study, a daily protein supplement of 21.2 g containing 6.2 g of leucine was also provided to both groups.^31^ The duration of n-3 PUFA supplementation ranged from 4 weeks to 6 months, with 4 studies supplementing for 8 weeks or less,^13^^,^^15^^,^^29^^,^^30^ and 4 studies supplementing for longer than 8 weeks.^28^^,^^31–33^ Placebos were used as controls in 7 studies, and these were corn oil,^13^^,^^28^^,^^30^ safflower oil,^32^ olive oil,^29^ sunflower oil,^15^ and an unspecified matching placebo.^33^ A protein supplement was used as a control in 1 study,^31^ 2 studies implemented a supervised period of resistance training,^30^^,^^32^ and 1 study implemented 2 weeks of leg immobilization.^15^

### Assessment of MPS and other outcomes

Of the 6 studies^13^^,^^15^^,^^28^^,^^30–32^ that expressed MPS as FSR, 2 measured mixed MPS (ie, all muscle proteins combined),^13^^,^^28^ and 4 studies measured myofibrillar MPS.^15^^,^^30–32^ One study calculated the absolute synthetic rate to quantify MPS.^30^ Additionally, whole-body protein synthesis was measured in 3 studies,^28^^,^^31^^,^^33^ and whole-body protein balance was calculated in 2 studies.^28^^,^^33^ A hyperaminoacidemic-hyperinsulinemic clamp was applied in 1 study to simulate a mixed macronutrient meal and both basal and stimulated FSRs were reported.^13^ One study administered a casein (0.06 g/kg fat-free mass) and 20 g Polycose product to mimic a fed state, reporting MPS under both fasted and fed conditions.^29^ In 3 studies, FSR was measured in response to an acute bout of exercise known to stimulate MPS.^17^^,^^28^^,^^33^ The remaining studies reported MPS in the unstimulated basal condition only.^31^^,^^33^^,^^15^ MPS was measured using the intravenous tracer infusion of l-(ring-^13^C6) phenylalanine in 3 studies,^13^^,^^28^^,^^29^ whereas 4 studies used deuterium oxide tracer methodology for the measurement of free-living integrates rates of MPS.^15^^,^^30–32^ The duration of MPS measurement (ie, tracer incorporation period) ranged from 1 hour to 2 weeks, depending on the protocol and choice of tracer.

Moreover, muscle strength was evaluated in 6 studies^15^^,^^28–32^ as handgrip strength,^29^^,^^31^ knee extension, and flexion torque.^15^^,^^28^^,^^30–32^ Muscle quality was assessed in 2 studies^28^^,^^32^ using a 1-repetition maximum per lean leg mass^28^ and knee extensor isometric strength per unit muscle cross-sectional area (CSA).^33^ Muscle mass was reported in 6 studies,^15^^,^^28–31^ as leg lean mass,^15^^,^^28^ appendicular lean mass,^29^^,^^31^ and thigh fat-free mass.^30^ Muscle CSA was measured in 3 studies; ^15^^,^^30^^,^^32^ 2 studies assessed muscle mass using magnetic resonance imaging of the quadriceps,^15^^,^^32^ while muscle fiber CSA was directly visualized and measured from muscle biopsy under fluoroscopy in another trial.^30^. Muscle volume was evaluated in 1 study^15^ from the sum of quadriceps CSA obtained from magnetic resonance imaging. Lean body mass was assessed in 2 studies.^29^^,^^30^ Finally, performance was measured in 4 studies,^28^^,^^29^^,^^31^^,^^32^ represented by peak oxygen consumption,^28^ short physical performance battery,^31^ 5 times sit to stand,^31^ timed up-and-go test,^31^ balance,^31^ physical activity score,^29^ quality of life score,^29^ maximal inspiratory pressure,^29^ gait speed,^31^^,^^32^ and chair-rise time.^32^

### Effect of n-3 PUFA supplementation on MPS

**Figure 2.**
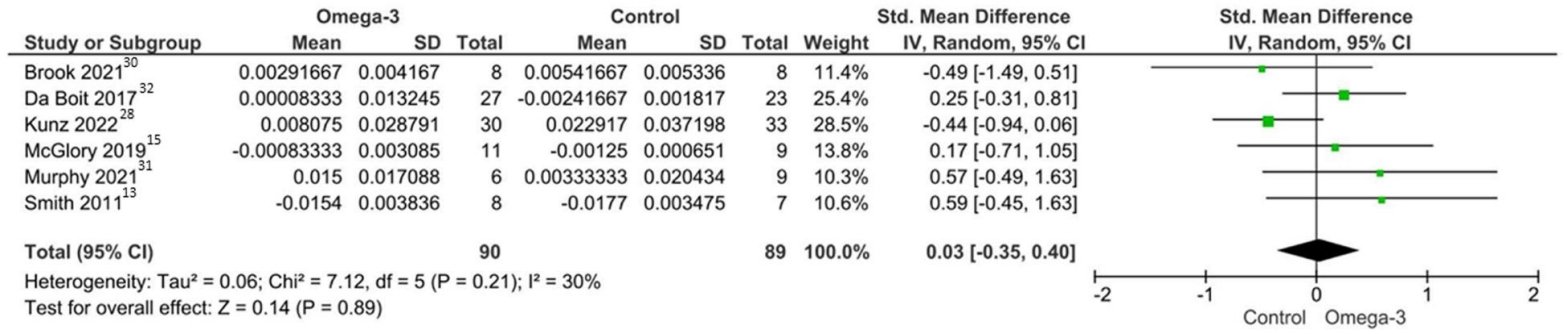
Effect of omega-3 polyunsaturated fatty acid supplementation on muscle protein synthesis. *Abbreviations*: df, degrees of freedom; IV, inverse variance; n-3 PUFA, omega-3 polyunsaturated fatty acid; SMD, standardized mean difference

Our main analysis (*k* = 6) revealed no effect of n-3 PUFA supplementation on MPS and low heterogeneity among included RCTs (SMD: 0.03; 95%CI, −0.35 to 0.40; *I*^2^ = 30%; *P* = .89) (Figure 2). In 1 study that reported both basal and stimulated FSR,^13^ basal FSR was selected for calculation of the pooled effect.

The results remained unchanged across subgroup analyses involving participants aged >50 years (*k* = 5; SMD: 0.02; 95%CI, −0.43 to 0.47; *I*^2^ = 42%; *P* = .93) (Figure S1). Subgroup analysis stratified by n-3 PUFA supplementation dose yielded similar results, with reduced heterogeneity with lower daily n-3 PUFA dosing strategies (*k* = 4; SMD: −0.05, 95%CI, −0.63–0.53, *I*^2^ = 45%, *P* = .87 for doses of n-3 PUFA supplementation >3 g/d; and *k* = 2, SMD: 0.23, 95%CI: −0.24 to 0.70, *I*^2^ = 0%, *P* = .34 for doses of n-3 PUFA supplementation <3 g/d) (Figure S2). Data from a shorter duration of n-3 PUFA supplementation (<8 weeks) demonstrated lower heterogeneity (*k* = 3; SMD: 0.08; 95%CI, −0.51 to 0.67; *I*^2^ = 10%; *P* = .78) compared with longer-duration supplementation despite similar overall effects (*k* = 3; SMD: 0.03; 95%CI, −0.55 to 0.62; *I*^2^ = 58%; *P* = .92) (Figure S3). Subgroup analysis involving studies with regular resistance exercise performed by both control and n-3 PUFA groups (*k* = 2, SMD: −0.06; 95%CI, −0.86 to 0.74; *I*^2^ = 51%; *P* = .88) and without additional regular training (*k* = 5; SMD: −0.03; 95%CI, −0.49–0.43; *I*^2^ = 32%; *P* = .89) did not reveal an increase in FSR (Figure S4).

A single study^13^ provided both basal FSR and stimulated (after a bout of resistance exercise) FSR values. Hence, we performed a subgroup analysis based on different conditions at which FSR was measured (without anabolic stimulation vs with anabolic stimulation). The results generated were similar for studies in which FSR was measured under fasting conditions with exercise bout to induce MPS (*k* = 3; SMD: 0.41; 95%CI, −0.16 to 0.98; *I*^2^ = 0%; *P* = .16), and for trials in which an anabolic stimulus was provided in relation to the measurement of MPS (*k* = 4; SMD: 0.52; 95%CI, −0.64 to 1.67; *I*^2^ = 77; *P* = .28) (Figure S5). In 1 study, FSR was reported under both basal and stimulated conditions.^13^ Different methods of FSR measurement also revealed no difference in FSR after n-3 PUFA supplementation and control for studies using oral deuterium oxide tracer methodology (*k* = 4; SMD: 0.16; 95%CI, −0.23 to 0.56; *I*^2^ = 0%; *P* = .42) and studies using the infusion of l-(ring-^13^C6)phenylalanine as the tracer (*k* = 2; SMD: −0.03; 95%CI, −1.02 to 0.95; *I*^2^ = 67; *P* = .95) (Figure S6). Analysis of studies that measured myofibrillar FSR (*k* = 4; SMD: 0.16; 95%CI, −0.23 to 0.56; *I*^2^ = 0%; *P* = .42) and mixed FSR (*k* = 2; SMD: −0.03; 95%CI, −1.02 to 0.95; *I*^2^ = 67; *P* = .95) also showed no difference in MPS values between n-3 PUFA and control or placebo groups (Figure S7).

No effect of n-3 PUFA ingestion on MPS was observed when a study that implemented 2 weeks of leg immobilization was removed from the analysis (*k* = 5; SMD: 0.02; 95%CI, −0.43 to 0.47; *I*^2^ = 42%; *P* = .93) (Figure S8). Moreover, when excluding a trial with leucine supplementation in both the intervention and control groups, no change in the pooled effect was observed (*k* = 5; SMD: −0.04; 95%CI, −0.43 to 0.36; *I*^2^ = 32%; *P* = .86) (Figure S9). Finally, after sensitivity analysis to exclude studies with a high risk of bias, no effect of n-3 PUFA supplementation on MPS was observed (*k* = 5; SMD: −0.03; 95%CI, −0.49 to 0.43; *I*^2^ = 32%; *P* = .89) (Figure S10).

### Effect of n-3 PUFA supplementation on whole-body protein synthesis

**Figure 3.**
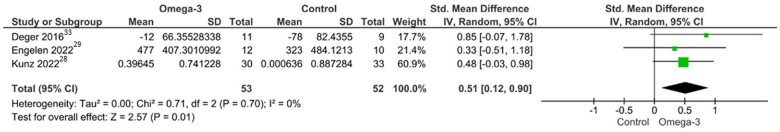
Effect of omega-3 polyunsaturated fatty acid supplementation on whole-body protein synthesis. *Abbreviations*: df, degrees of freedom; IV, inverse variance; n-3 PUFA, omega-3 polyunsaturated fatty acid; SMD, standardized mean difference

Our main analysis (*k* = 3) revealed that whole-body protein synthesis rates significantly increased after n-3 PUFA supplementation compared with placebo (*k* = 3; SMD: 0.51; 95%CI, 0.12–0.90; *I*^2^ = 0%; *P* = .01) (Figure 3). Included trials exhibited low heterogeneity. In 1 study, 2 doses of n-3 PUFA supplementation were administered at the equivalents of 2 g/d and 3.5 g/d. In our main analysis, participants received 3.5 g/d n-3 PUFA, given that this dose is closer to that used in other studies included in the meta-analysis. However, when studies in which 2 g/d n-3 PUFA was administered were included in the analysis, the result remained the same (*k* = 3; SMD: 0.48; 95%CI, 0.09–0.88; *I*^2^ = 0%; *P* = .02) (Figure S11). Two of the included studies were performed with individuals with a comorbidity (namely, COPD^29^ and end-stage renal disease treated with maintenance hemodialysis^33^). Hence, subgroup analysis based on participants’ health status (healthy vs with comorbidities) revealed insignificant results with a trend toward greater whole-body protein synthesis via n-3 PUFA supplementation (*k* = 2; SMD: 0.51; 95%CI, −0.06 to 1.20; *I*^2^ = 0%; *P* = .07) (Figure S12).

### Risk of bias assessment

**Figure 4.**
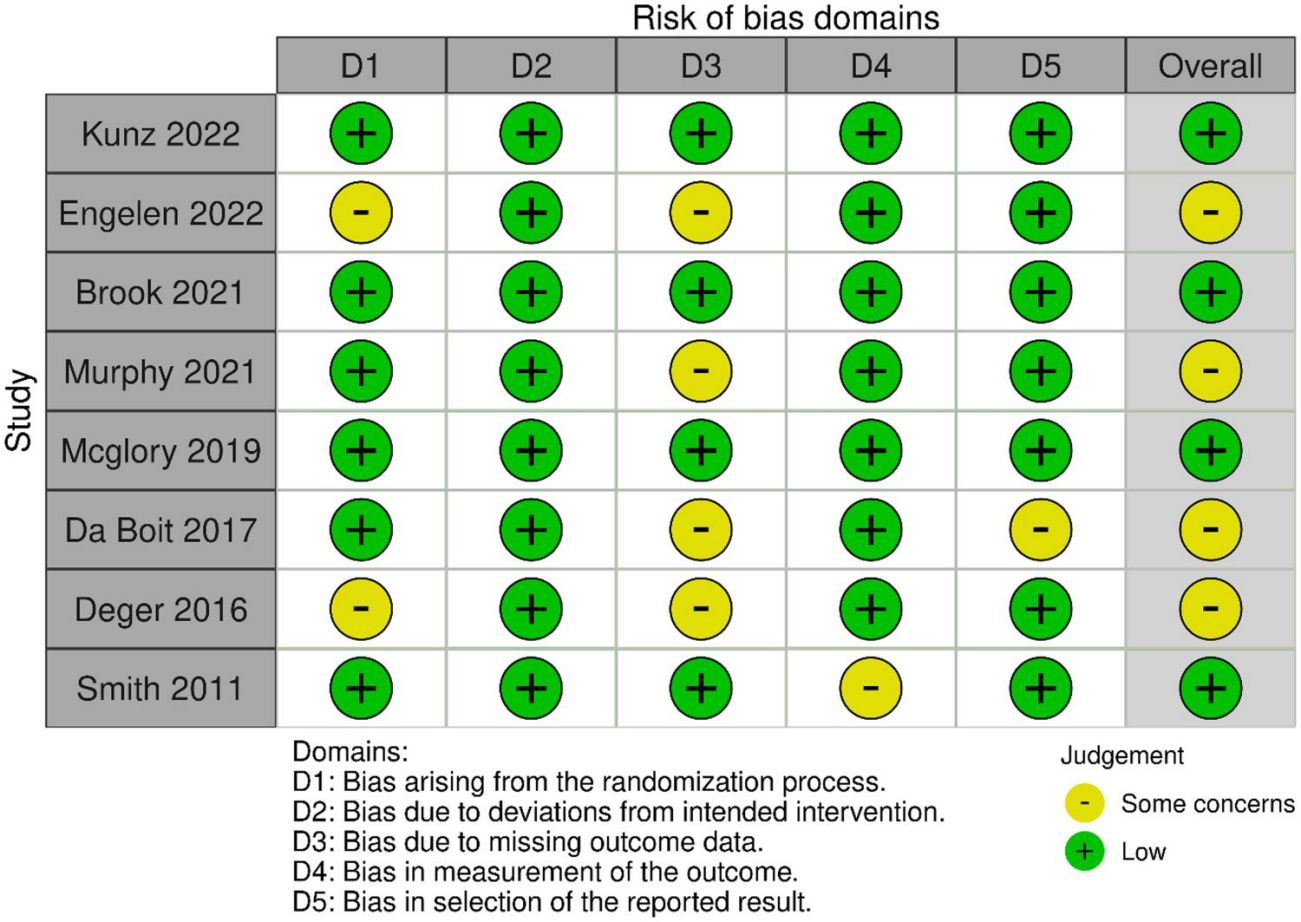
Quality assessment of the included studies based on the Cochrane risk-of-bias tool for randomized trials

Risk of bias findings according to the RoB2 tool are presented in Figure 4.

### Publication bias

Because of the limited number of studies in the meta-analysis, a funnel plot and Egger’s test were not performed (k < 10).

## DISCUSSION

This systematic review and meta-analysis determined the effect of n-3 PUFA supplementation on MPS and whole-body protein synthesis rates in healthy adults and clinical populations. Overall, we report a significant increase in whole-body protein synthesis rates with n-3 PUFA supplementation, with 3 studies reporting a trend for increased whole-body protein synthesis rates.^28^^,^^29^^,^^33^ Conversely, we observed no significant effect of n-3 PUFA supplementation on MPS. The lack of effect of n-3 PUFA ingestion on MPS was underscored by the relatively low number of participants recruited to each trial and the relatively large individual variation in MPS response by control and intervention groups.^33^ Our data suggest that n-3 PUFA supplementation is more beneficial in individuals with chronic inflammation and cachexia compared with healthy adults. Indeed, the preponderance of studies included in the meta-analysis that evaluated whole-body synthesis rates recruited clinical population groups with comorbidities.^30^^,^^33^ Indeed, our subgroup analysis in participants with comorbidities showed a trend toward higher whole-body protein synthesis rates when supplemented with n-3 PUFA.

In contrast to our measurement of whole-body protein synthesis rates, our main analysis (*k* = 6) revealed no significant effect of n-3 PUFA ingestion on MPS rates regardless of participant age, dose, and duration of supplementation, resistance exercise training, and method of FSR measurement. Interestingly, a series of recent meta-analyses reported a small, but clinically relevant, increase in skeletal muscle mass,^34^ lower-body strength,^35^ and muscle function (as measured by improved timed up-and-go test and sit-to-stand test)^34^^,^^35^ after n-3 PUFA supplementation compared with a control in both healthy adults and clinical populations. Notably, several of the studies analyzed in our systematic review reported an increase in skeletal muscle mass in the absence of significant changes in MPS. These findings suggest that n-3 PUFA supplementation elicits a beneficial effect on musculoskeletal health by improving muscle mass and function. However, based on our findings, the causal mechanism of action was not independently attributed to an increased stimulation of MPS.

Although n-3 PUFA ingestion might not augment basal rates of MPS, it is suggested that n-3 PUFA ingestion increases the response of MPS to anabolic stimuli. Accordingly, Smith et al^13^^,^^14^ demonstrated that n-3 PUFA supplementation did not alter basal MPS rates compared with placebo; however, n-3 PUFA supplementation augmented the MPS response to ahyperaminoacidemia-hyperinsulinemia stimulus and increased the phosphorylation status of mTORC-1 signaling protein in young and older adults. Similarly, Engelen et al^29^ reported that high-dose n-3 PUFA supplementation enhanced feeding-induced net protein synthesis rates without modulating basal protein anabolism. Moreover, although older adults exhibit a less pronounced transcriptional response to exercise regarding protein synthesis compared with their younger counterparts,^27^ the difference was reduced after 4 months of n-3 PUFA supplementation, with a downregulation in expression of negative regulators of muscle growth and protein synthesis such as myostatin and foxo1.

In our main analysis, basal unstimulated rates of MPS, as opposed to MPS rates measured after anabolic stimulation, were used to calculate the pooled effect in 50% of included studies. To explore the effect of n-3 PUFA ingestion on basal and stimulated rates of MPS, we performed a subgroup analysis based on the conditions of MPS measurements (basal vs stimulated by feeding or acute exercise). However, we did not detect any increase in MPS in response to anabolic stimulation with n-3 PUFA ingestion. Although Smith et al^13^ measured MPS within 2.5 hours of anabolic stimulation by hyperaminoacidemia-hyperinsulinemia clamp, other studies used exercise as the anabolic stimulus. Moreover, the timing of muscle biopsies ranged from 1 h to 4 h after exercise. One possible explanation for the lack of effect of n-3 PUFA ingestion in the studies with acute exercise stimulation is that the bout of acute exercise was not of sufficient intensity or volume to stimulate MPS in anabolic-resistant individuals.^36^^,^^37^ Conversely, intense exercise can trigger MPS to a great extent, reaching a point where further improvement is not possible; this is known as a plateau effect.^38^ Hence, additional intake of n-3 PUFA does not enhance MPS. For instance, in young men who underwent resistance exercise training, whey protein supplementation did not increase MPS.^16^ Similarly, in older adults, combining leucine-enriched protein with n-3 PUFA did not result in any additional response of MPS compared with protein supplementation alone.^33^ Given this potential plateau effect, it is conceivable that n-3 PUFA ingestion is more beneficial in clinical populations than in healthy individuals.

The notion that n-3 PUFA supplementation attenuates MPB rather than upregulates MPS provides a logical explanation for why we could not detect a change in FSR with n-3 PUFA ingestion.^39^ Consistent with this notion, Engelen et al^29^ reported a decrease in net protein breakdown during the fasted state after 3.5 g/d n-3 PUFA ingestion in 10 patients with COPD. Similarly, Deger et al^33^ reported a decreased response of MPB in patients undergoing hemodialysis who received 12 weeks of n-3 PUFA supplementation. The anti-inflammatory properties of n-3 PUFA are widely established. Accordingly, an umbrella meta-analysis reported reduced circulating C-reactive protein, tumor necrosis factor-α, and interleukin-6 concentrations in healthy and diseased elderly participants who received n-3 PUFA supplementation.^40–43^ Our recent meta-analysis also confirmed the reduction in tumor necrosis factor-α and interleukin-6 in patients with heart failure, after n-3 PUFA ingestion.^44^ The ingestion of n-3 PUFA was also shown to reduce inflammation-induced muscle damage in parallel to the reduction of inflammatory cytokines.^45^ The anti-inflammatory effects of n-3 PUFA are due to its ability to displace arachidonic acid on circulating lipids^46^ and cell membranes,^47^ leading to the production of eicosanoids with lower inflammatory potency.^48^ Additionally, n-3 PUFA can compromise cyclooxygenase-2 production by preventing nuclear binding of nuclear factor κB^49^ through activation of peroxisome proliferator-activated receptor γ^50^^,^^51^ and stimulation of G-protein coupled receptor.^48^ n-3 PUFA can be converted into proresolution mediators that can reduce inflammatory cytokine production. Additionally, the anti-inflammatory effect of n-3 PUFAs can indirectly inhibit MPB via the downregulation of nuclear factor-κB and downstream inhibition of the muscle ring finger-1 (MuRF-1) gene. In this regard, the MuRF-1 gene encodes for the rate-limiting enzyme of ubiquitin–proteasome system as the main cellular protein breakdown machinery. This finding was confirmed by a suppressed expression of ubiquitin-mediated proteolysis from microarray analysis after long-term n-3 PUFA supplementation.^52^ Moreover, preliminary data exists that suggest n-3 PUFA supplementation facilitates mitochondrial function, leading to a 20%–25% reduction in the production of reactive oxygen species^53^^,^^54^ and decreased susceptibility to oxidative damage.^55^ n-3 PUFA also increases expression of key electron transport proteins in the mitochondria.^52^^,^^56–59^ Although studies have not consistently measured MPB, preliminary evidence suggests that n-3 PUFA ingestion elicits a decrease in MPB. Therefore, n-3 PUFA might be more beneficial in disease conditions with protein-energy wasting or cachexia.

The lack of significant improvement in MPS in the pooled results can also be explained by variation in individual responses to n-3 PUFA supplementation. Several studies indicate that women might respond better to n-3 PUFA supplementation than men.^17^^,^^27^ Women exhibited a greater propensity to synthesize eicosapentaenoic acid and docosahexaenoic acid than do men.^60^ This sexual dimorphism also might be attributed to the differential enrichment of n-3 PUFA in the cell membrane after n-3PUFA supplementation.^34^ Moreover, women responded less to resistance exercise than did men, which limited improvements in muscle strength and quality after resistance exercise.^61^ Therefore, the effect of n-3 PUFA when combined with resistance exercise is likely more pronounced in women, given the greater capacity for improvement in muscle health–related outcomes. Other authors have suggested that higher baseline levels of inflammation would allow anti-inflammatory effects of n-3 PUFA to result in larger musculoskeletal benefits.^31^ However, to our knowledge, no study has identified any characteristics, phenotypes, or biomarkers that clearly distinguish responders and nonresponders to n-3 PUFA ingestion in terms of muscle health–related outcomes.^27^

To date, to our knowledge, no RCT has investigated the effect of n-3 PUFA ingestion on MPS in patients with sarcopenia. A seminal study by Smith et al^13^ demonstrated that n-3 PUFA supplementation augmented the MPS response to anabolic stimuli by ∼30% in healthy older adults with mean age of 71 years. In contrast, a recent RCT with healthy older adults whose mean age was 69–71 years revealed no significant change in MPS after ingesting 3 g/d n-3 PUFA over 18 weeks.^17^ No increases in FSR and whole-body protein synthesis were observed in healthy older adults after 4 g/d n-3 PUFA ingestion over 6 months.^29^ However, that study reported significant improvement in muscle strength in participants who received n-3 PUFA compared with placebo.

Trials with more frail elderly people provide more promising results with regard to the anabolic effect of n-3 PUFA ingestion. Accordingly, an ∼15% increase in net protein synthesis was observed after 4 weeks of n-3 PUFA supplementation in a cohort of patients with COPD whose mean age was 67 years, whereas net protein breakdown decreased by 13%^31^ and whole-body protein synthesis rates remained unchanged with n-3 PUFA supplementation. Moreover, Deger et al^33^ conducted their study with patients undergoing hemodialysis who had chronic inflammation, a condition generally leading to muscle loss. The results yielded a nonsignificant trend toward greater whole-body protein synthesis after 12 weeks of n-3 PUFA supplementation and a significant reduction of MPB. When the data are pooled, n-3 PUFA supplementation significantly augmented whole-body protein synthesis.

### Strength and limitations

To our knowledge, this meta-analysis is the first to investigate the effect of n-3 PUFA supplementation on rates of MPS and whole-body protein synthesis. Nonetheless, our meta-analysis was limited by the increased heterogeneity pertinent to the methods used for the measurement of MPS. To minimize the effect of variation in the measurement protocol, we performed subgroup analysis–based isotope tracer methodology, fraction-specific measurements of MPS and the conditions (ie, basal vs stimulated) under which MPS were determined. However, discrepancies still exist in terms of duration of MPS measurement and the type (exercise or nutrition) and timing of anabolic stimulation. Irrespective of whether subgroup analyses were performed, n-3 PUFA supplementation protocols (dose and duration) and resistance training variables were not standardized across studies. Moreover, the dose and formula of n-3 PUFA supplementation differed between studies; several supplements contained vitamin D and other lipids to physiologically relevant concentrations, which might affect n-3 PUFA bioavailability.^62^ Although we have assigned the random-effects model in our calculation, these discrepancies could still lead to heterogeneity of results. Because of limited number of studies and few participants in each study, the pooled sample size was small (N = 188; n = 90 in n-3 PUFA group and n = 98 in control group). Some subgroup analyses only contained 2 studies, limiting reliability. Moreover, the analysis of whole-body protein synthesis rates should be interpreted with caution given that only 3 studies were included with a total of 105 participants (n = 53 in the n-3 PUFA group, n = 52 in the control group), 2 of whom were in individuals with end-stage kidney disease receiving hemodialysis or with COPD. Therefore, extrapolation to a general elderly population needs thorough consideration.

### Future research

Moving forward, larger RCTs are required to investigate the effect of n-3 PUFA ingestion on whole-body and tissue-specific (muscle) protein synthesis rates. In addition, these studies should also measure MPB to clarify the underlying mechanism of the musculoskeletal benefits of n-3 PUFA. To determine the effects of n-3 PUFA on anabolic resistance, both basal FSR and stimulated FSR after exercise or feeding should be measured and compared before and after n-3 PUFA supplementation and placebo administration. Studies comparing the response of MPS with n-3 PUFA ingestion in healthy individuals vs patient groups with chronic wasting conditions are warranted.

## CONCLUSION

This systematic review and meta-analysis revealed that n-3 PUFA ingestion has the potential to enhance whole-body protein synthesis rates, although no significant effects were observed in terms of MPS. These conflicting findings were attributed, at least in part, to variations in n-3 PUFA supplementation regimens and measurement protocols for the assessment of MPS. Moreover, the beneficial effect of n-3 PUFA ingestion on muscle health may extend beyond MPS, with preliminary evidence linking n-3 PUFA ingestion with improved neuromuscular function. Notwithstanding, although n-3 PUFA ingestion is a safe and affordable nutritional intervention, further investigation is necessary to yield a deeper understanding of the mechanisms that underpin any musculoskeletal health benefits of n-3 PUGA ingestion.

## Supplementary Material

nuae055_Supplementary_Data

## Data Availability

Data are available upon request.
