# Supplementary material for: The effects of omega-3 polyunsaturated fatty acids on muscle and whole-body protein synthesis: a systematic review and meta-analysis
Source: Nutr Rev. 2024 May 23;83(2):e131–43. doi: 10.1093/nutrit/nuae055 (PMC11723138; doi:10.1093/nutrit/nuae055)

Figure S1. Effect of omega-3 supplementation on muscle protein synthesis in older adults 50 years or above.


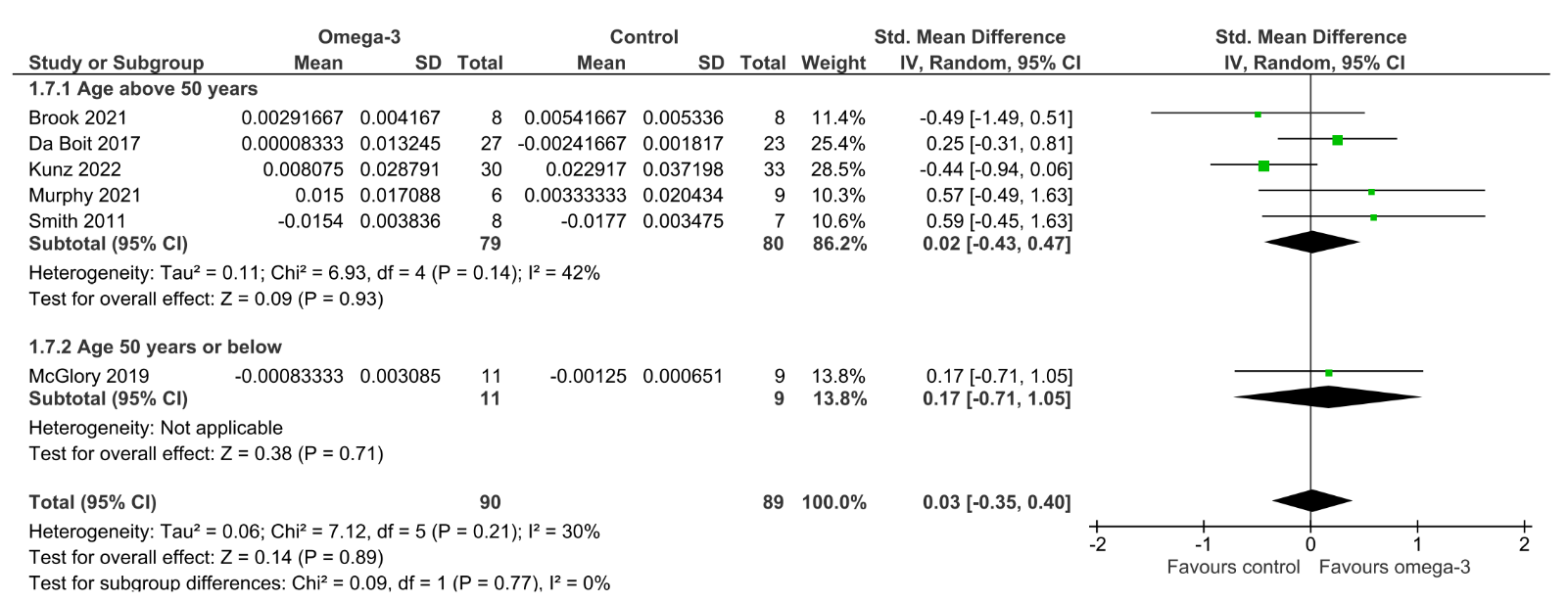


Figure S2. Effect of omega-3 supplementation on muscle protein synthesis based on treatment dose.


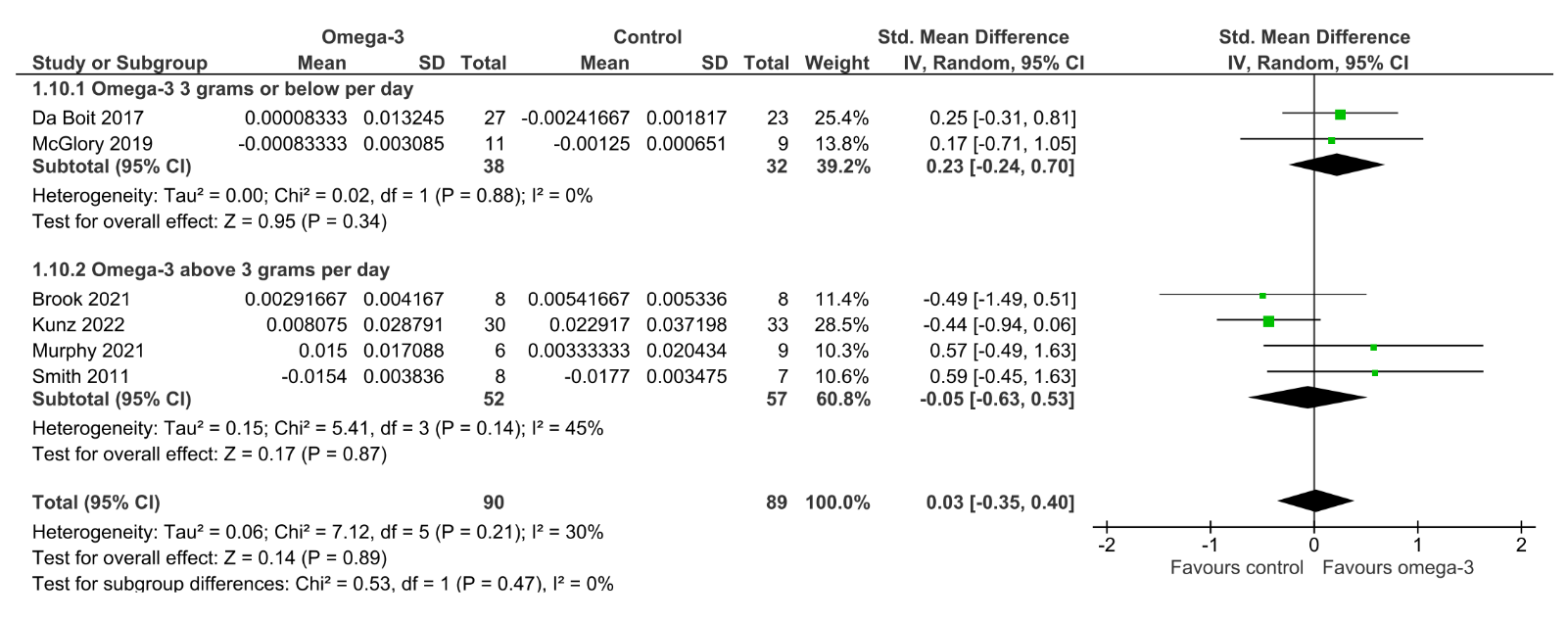


Figure S3. Effect of omega-3 supplementation on muscle protein synthesis based on treatment duration.


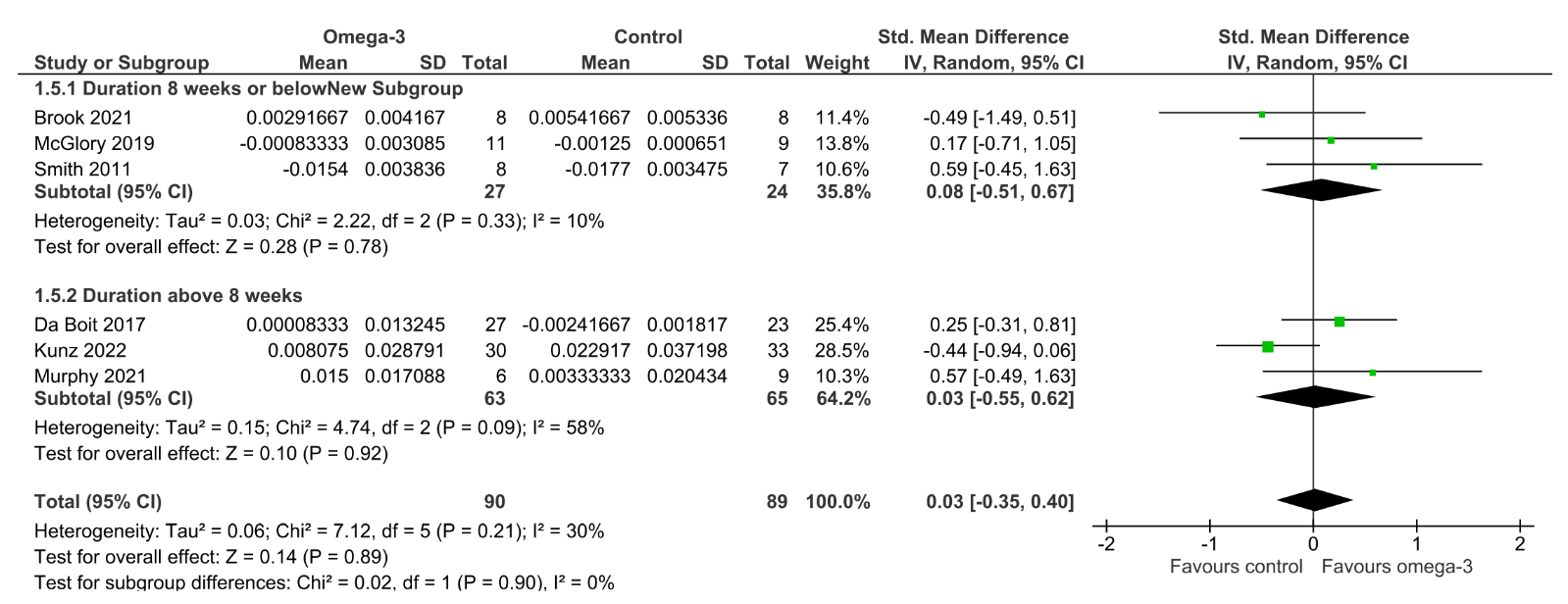


Figure S4. Effect of omega-3 supplementation on muscle protein synthesis based on the presence of regular resistance training.


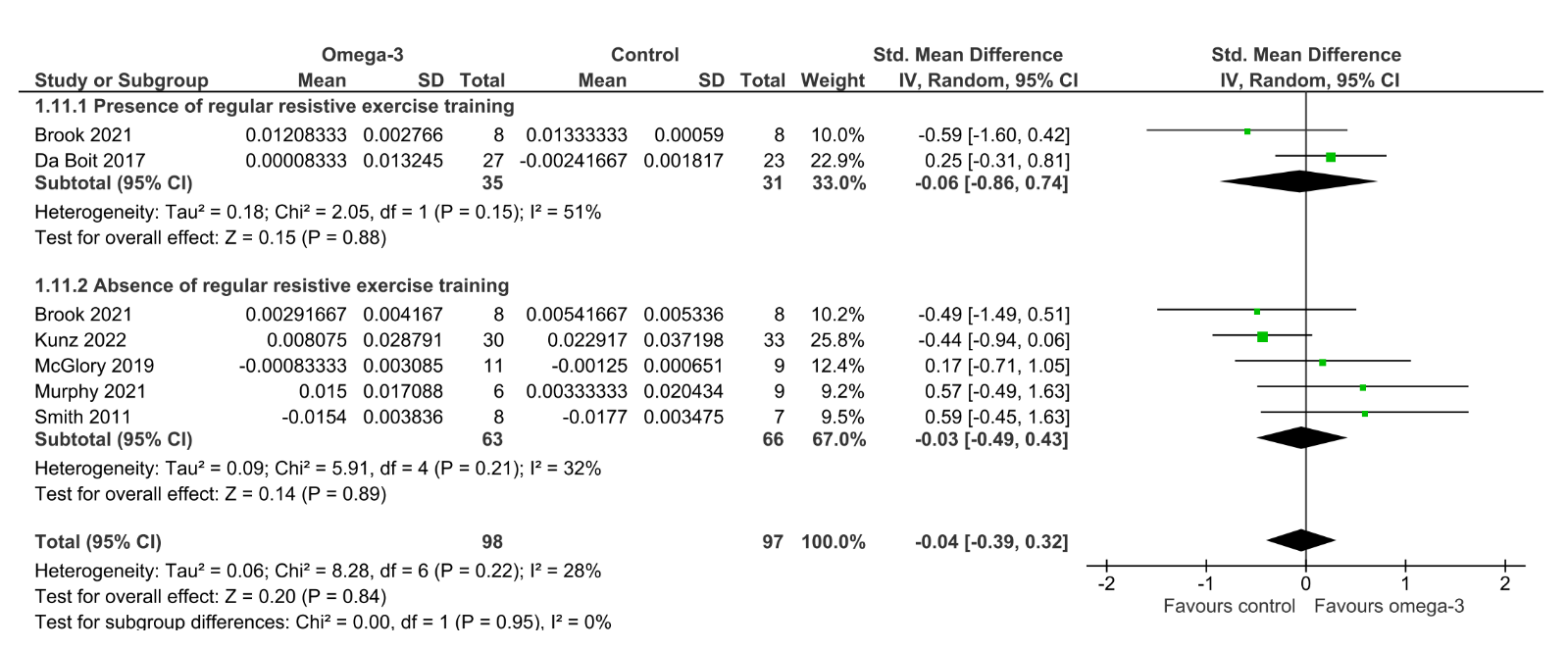


Figure S5. Effect of omega-3 supplementation on muscle protein synthesis based on the condition of fractional synthetic rate measurement


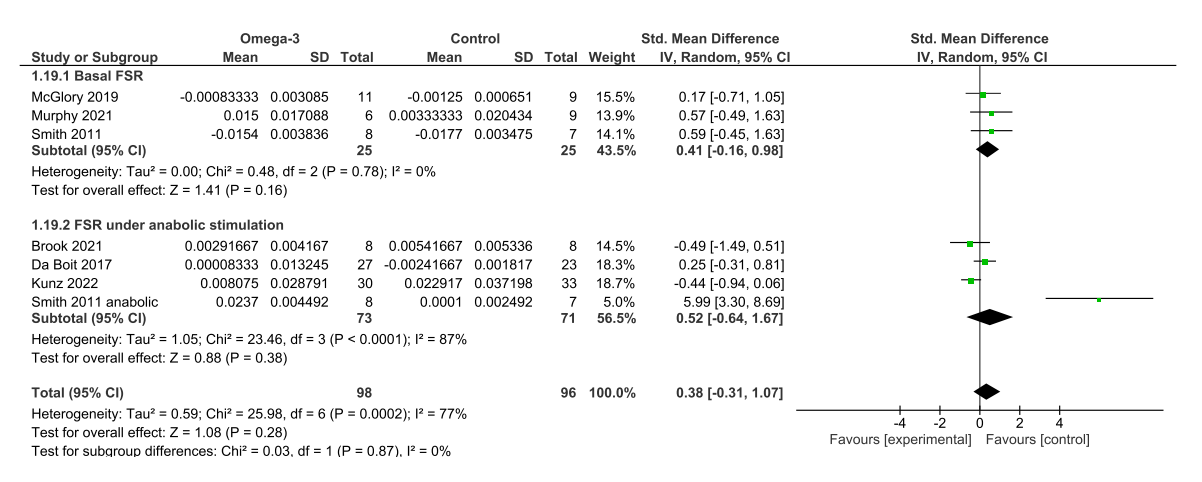

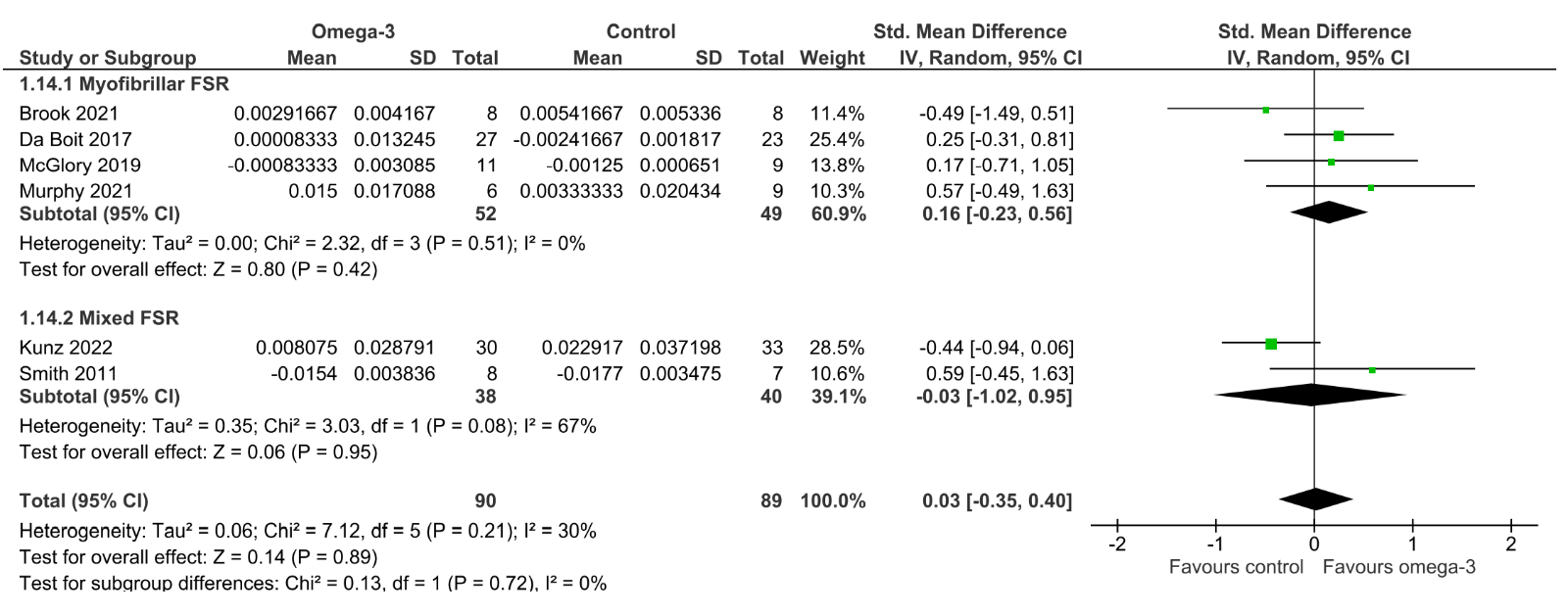


Figure S6. Effect of omega-3 supplementation on muscle protein synthesis based on types of amino acid tracers.


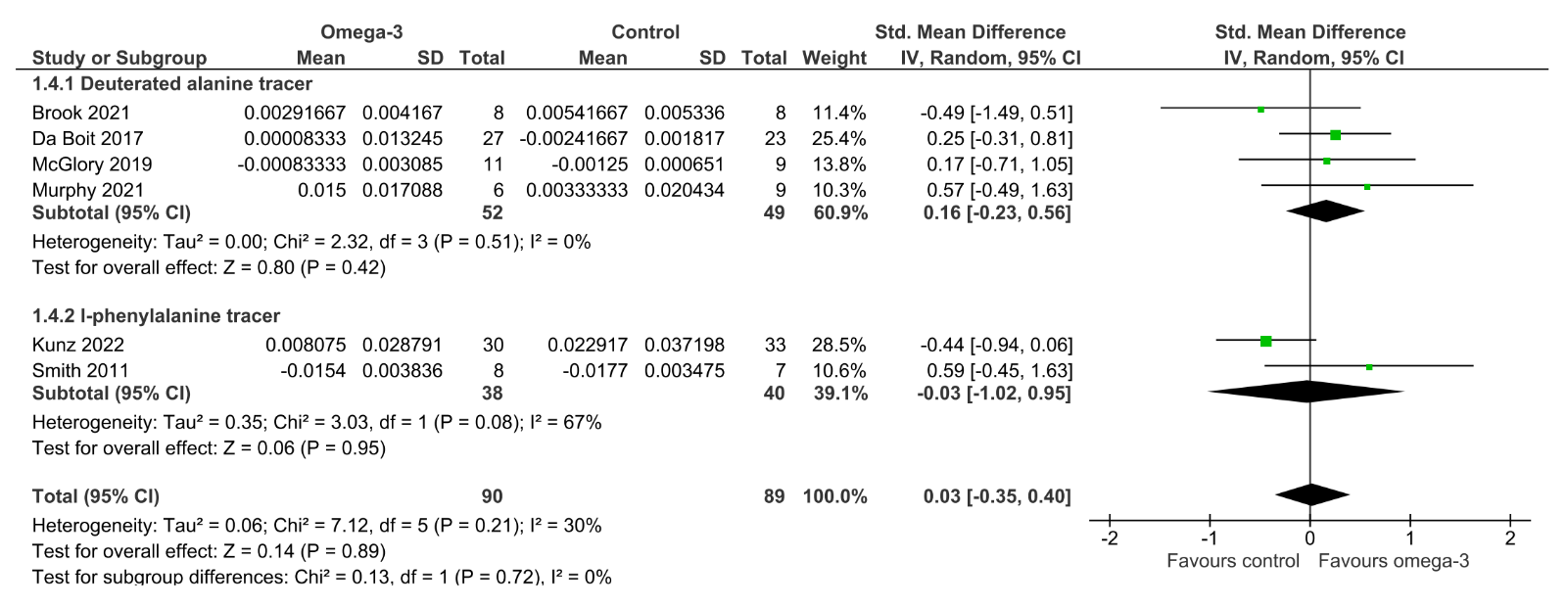


Figure S7. Effect of omega-3 supplementation on muscle protein synthesis based on the compartment of muscle protein synthesis assessment.


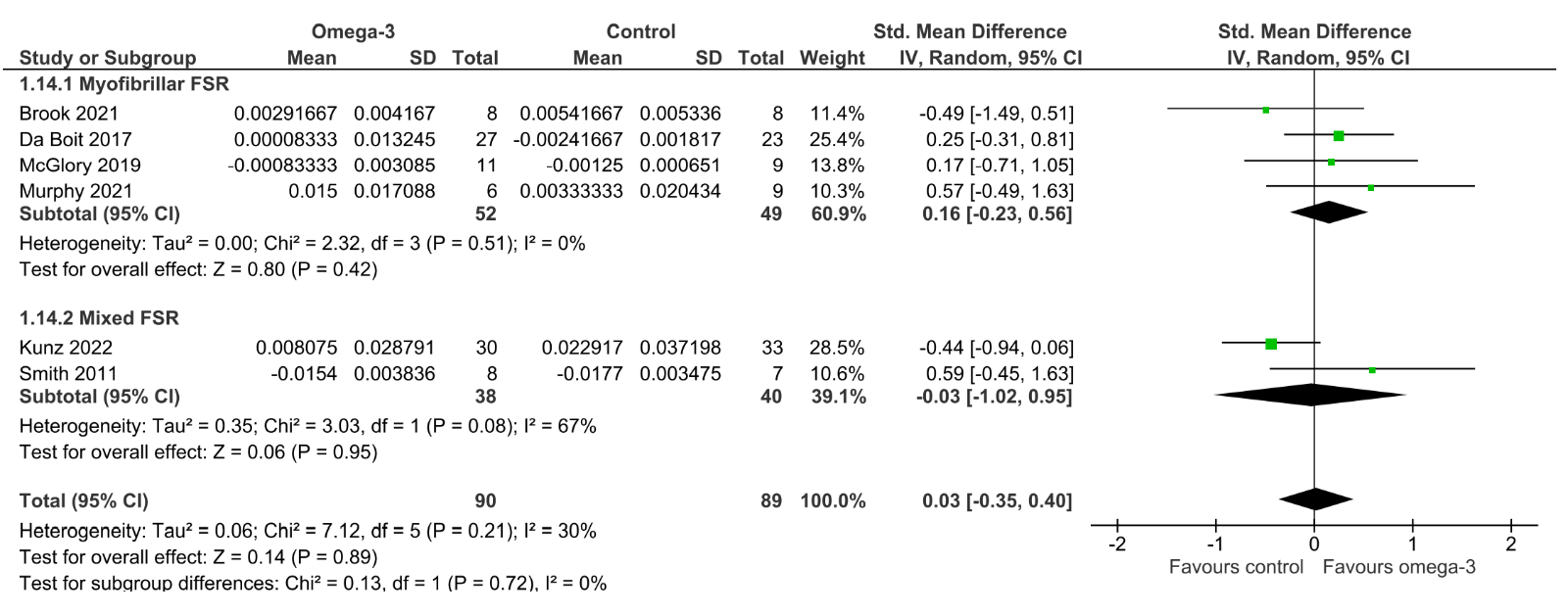


Figure S8. Effect of omega-3 supplementation on muscle protein synthesis excluding studies with leg immobilization.


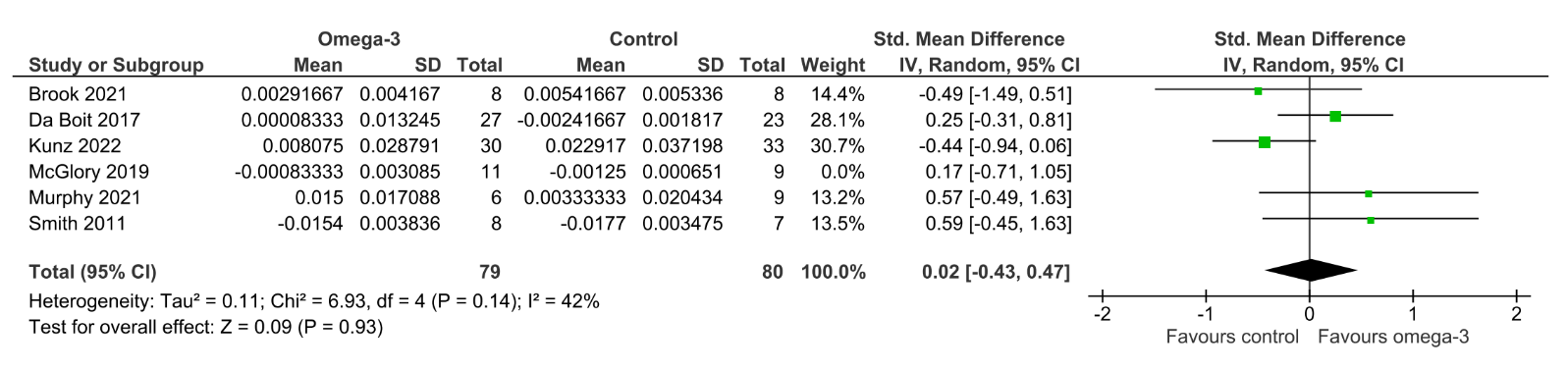


Figure S9. Effect of omega-3 supplementation on muscle protein synthesis based on studies without additional leucine supplementation.


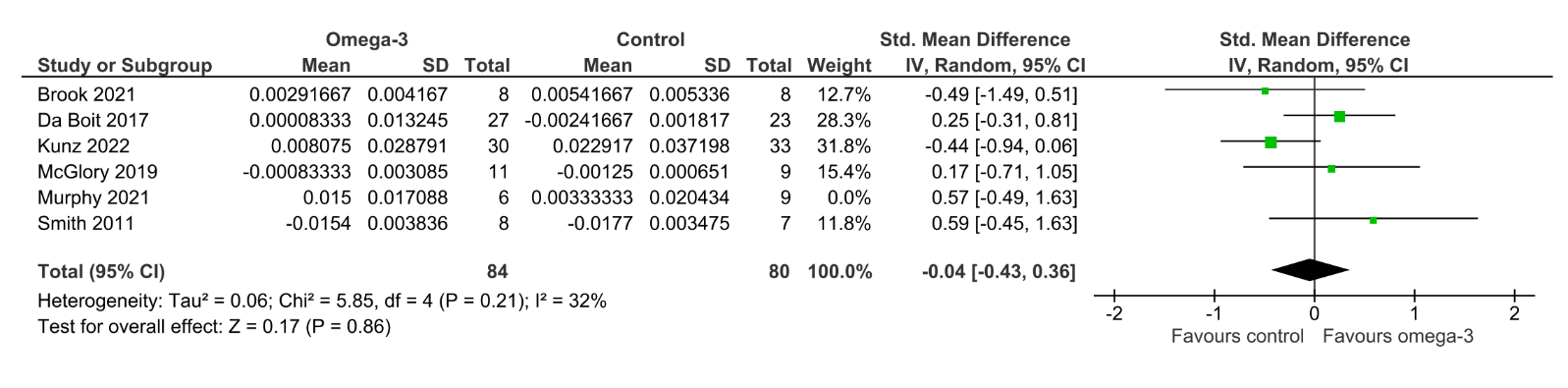


Figure S10. Effect of omega-3 supplementation on muscle protein synthesis based on risk of bias.


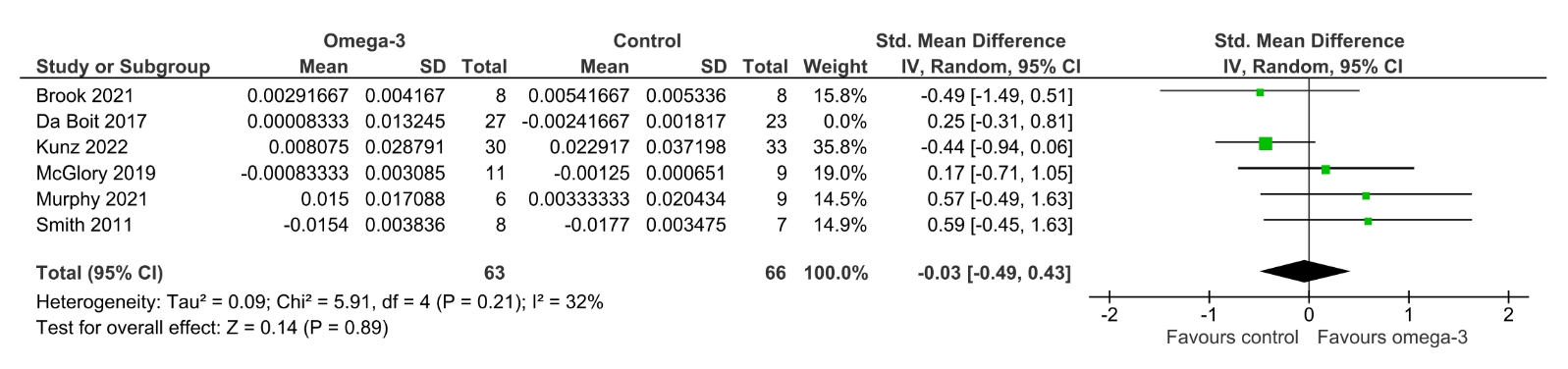


Figure S11. Effect of omega-3 supplementation on whole body protein synthesis with lower omega-3 dose.


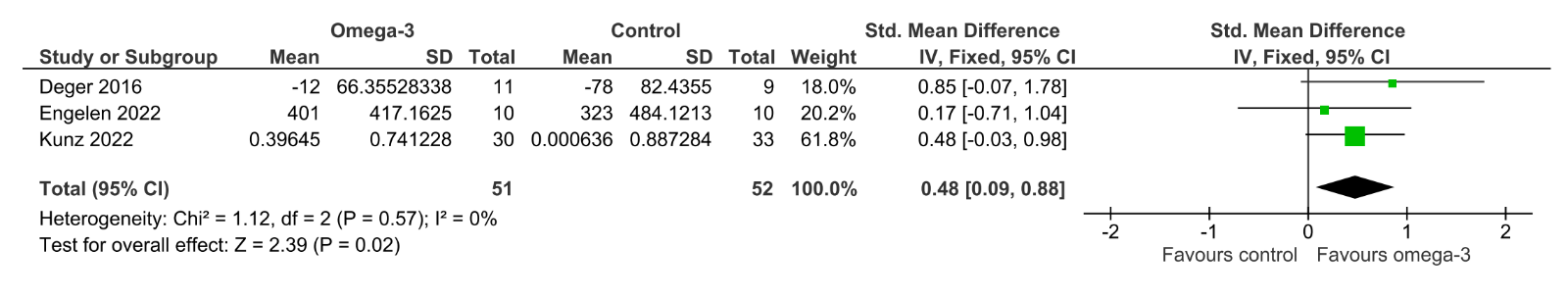


Figure S12. Effect of omega-3 supplementation on whole body protein synthesis based on participants’ health status


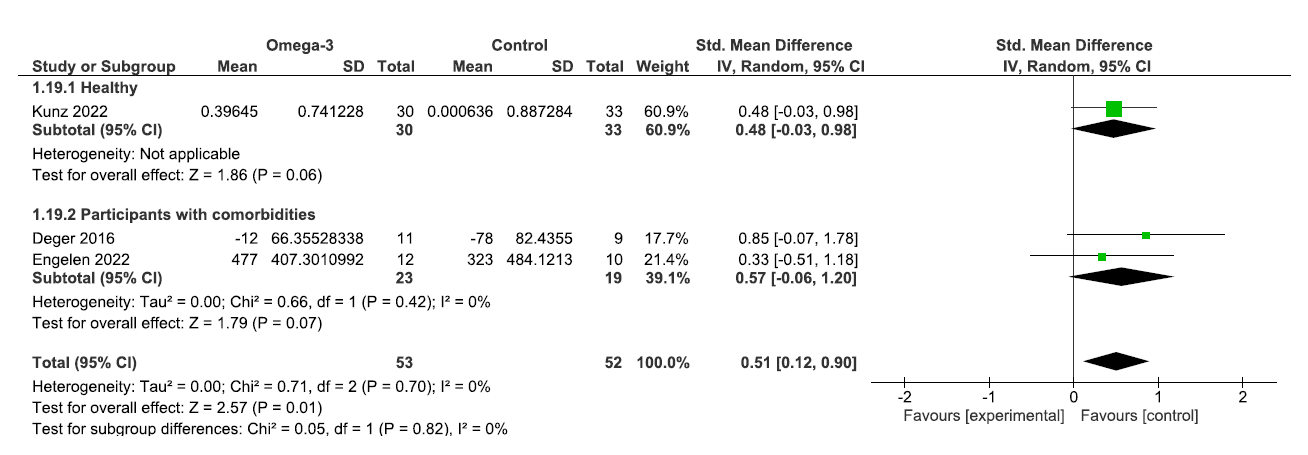

Supplement: nuae055_Supplementary_Data [file nuae055_supplementary_data.zip › nuae055_Supplementary_Data/Supplementary Figures.docx]
