# Supplementary material for: The effects of omega-3 polyunsaturated fatty acids on muscle and whole-body protein synthesis: a systematic review and meta-analysis
Source: Nutr Rev. 2024 May 23;83(2):e131–43. doi: 10.1093/nutrit/nuae055 (PMC11723138; doi:10.1093/nutrit/nuae055)
Supplement: nuae055_Supplementary_Data [file nuae055_supplementary_data.zip › nuae055_Supplementary_Data/Table S1.docx]

**Table S1.**Full search strategy protocol based on title, abstract, and keywords in the literature search.

| **Database** | **Search terms** |
| --- | --- |
|  |  |
| PubMed | (omega-3 fatty acids OR omega-3 polyunsaturated fat* OR fish oil OR eicosapentaenoic acid OR docosahexaenoic acid) AND  (Muscle protein synthesis OR whole-body protein synthesis OR fractional synthetic rate) |
| Cochrane Library | (omega-3 fatty acids OR omega-3 polyunsaturated fat* OR fish oil OR eicosapentaenoic acid OR docosahexaenoic acid) AND  (Muscle protein synthesis OR whole-body protein synthesis OR fractional synthetic rate) |
| Web of Science | (omega-3 fatty acids OR omega-3 polyunsaturated fat* OR fish oil OR eicosapentaenoic acid OR docosahexaenoic acid) AND  (Muscle protein synthesis OR whole-body protein synthesis OR fractional synthetic rate) |
| Scopus | (omega-3 OR eicosapentaenoic AND acid OR docosahexaenoic AND acid) AND (muscle AND protein AND synthesis OR  whole-body AND protein AND synthesis) |
