# Supplementary material for: The effects of omega-3 polyunsaturated fatty acids on muscle and whole-body protein synthesis: a systematic review and meta-analysis
Source: Nutr Rev. 2024 May 23;83(2):e131–43. doi: 10.1093/nutrit/nuae055 (PMC11723138; doi:10.1093/nutrit/nuae055)
Supplement: nuae055_Supplementary_Data [file nuae055_supplementary_data.zip › nuae055_Supplementary_Data/Table S2.docx]

**Table S2.** Employed PICOS framework.

| Participants | Any health status above 18 years of age |
| --- | --- |
| Intervention | Omega-3 fatty acid supplementation |
| Comparator | No restrictions: For the meta-analysis a placebo control was used. |
| Outcomes | Muscle and whole-body protein synthesis |
| Study design | Randomized and non-randomized controlled trials |
